# Supplementary material for: Hepatitis E and Allogeneic Hematopoietic Stem Cell Transplantation: A French Nationwide SFGM-TC Retrospective Study
Source: Viruses. 2019 Jul 5;11(7):622. doi: 10.3390/v11070622 (PMC6669459; doi:10.3390/v11070622)

Supplementary table 1: Follow-up of patients diagnosed after alloHSCT

Supplementary table 1A: Patients not treated

Patient	Time from Tx to testing	Time from Tx to HEV diagnosis	Time from HEV diagnosis						
			D+15	D+30	D+45	M3	M6	M9	Last news
**2**		D+4177							M+18
**Blood NAT**	Nd	Pos				Neg	Neg	Neg	Neg
**IgG/IgM**	Nd	Pos/Pos				Pos/Pos	Pos/Pos	Pos/Pos	
**4**		D+2895							M+20
**Blood NAT**	Nd	Pos			Neg				Neg
**IgG/IgM**	Nd	Pos/Pos			Pos/Pos				Pos/Pos
**5**	D-15	D+187							
**Blood NAT**	Nd	5.63				Neg	Neg		
**IgG/IgM**	Neg/Neg					Pos/Pos	Pos/Neg		
**6**	D-30	D+615							
**Blood NAT**	Neg	6.34	3.37				Neg		
**IgG/IgM**	Neg/Neg	Pos/Pos					Pos/Pos		
**8**		D+142							M+22
**Blood NAT**	Nd	Pos				Neg	Neg		Neg
**IgG/IgM**	Nd	Pos/Pos				Neg/Neg	Pos/Pos		Pos/Neg
**9**		D+148							
**Blood NAT**	Nd	Pos		Neg				Neg	
**IgG/IgM**	Nd	Neg/Neg		Neg/Neg			Neg/Neg	Neg/Neg	
**11**		D+280							
**Blood NAT**	Nd	Pos	Pos		Pos	Pos	Pos	Neg	
**IgG/IgM**	Nd	Nd	Nd		Nd/Pos	Nd	Nd	Nd	
**17**	M-1	D+1028							
**Blood NAT**	Nd	4.49	2.83		Neg	Neg			
**IgG/IgM**	Neg/Neg	Pos/Pos							
**23**		D+78							
**Blood NAT**	Nd	Pos	Pos	Pos	Pos		Neg	Neg	
**IgG/IgM**	Nd	Nd							

Abbreviations: Tx: transplantation; D: day; M: month; Pos: positive; NAT: nucleic acid testing; Nd: not done, Neg: negative. HEV NAT is indicated in log.

Supplementary table 1B: Patients treated with ribavirin

Pat	Time from Tx to testing	Time from Tx to HEV diagnosis	Time from HEV diagnosis to Ribavirin	Time from ribavirin initiation											
				D0	D+15	D+30	D+45	D+60	M3	M6	M7	M9	M12	M14	last news
**1**	D+30	D+126	D+11	Ribavirin: 175 d											
**Blood NAT**	Neg	5.57		3.5					<1	Neg		Neg	Neg		
**IgG/IgM**	Neg/Neg	Neg/Pos		Neg/Pos					Neg/Pos	Neg/Pos		Pos/Pos	Pos/Neg		
**3**	D-60	D+287	D+14	Ribavirin: 90 d (neutropenia)											
**Blood NAT**	Nd	Pos		5.9	2.5	Neg			Neg						
**IgG/IgM**	Neg/Neg	Neg/Pos		Neg/Pos	Neg/Pos	Neg/Pos			Pos/Pos						
**7**		D+1746	D+18	Ribavirin: 44 d											
**Blood NAT**	Nd	Pos		Pos			Nd		Neg						
**IgG/IgM**	Nd	Nd		Nd			Nd		Pos/Pos						
**10**	D+650	D+810	D+26	Ribavirin: 60 d (erythroblastopenia)							Ribavirin: 11 months				
**Blood NAT**	Neg	5.4		7.3					4.2	Pos		Neg	Neg		M18<br>Neg
**IgG/IgM**	Nd/Neg														
**12**		D+386	D+47	Ribavirin: 99 d											
**Blood NAT**	Nd	Pos		Pos					Neg	Neg			Neg		
**IgG/IgM**	Nd	Pos/Pos													
**13**	M-9	D+205	D+183	Ribavirin: 167 d											
**Blood NAT**	Pos	Pos		Pos		Pos			Pos		Neg	Pos	Pos	Pos	M20<br>Neg
**IgG/IgM**	Pos/Pos	Neg/Pos												Pos/Pos	
**14**	M-1	D+386	D+36	Ribavirin: 60 d (pancytopenia)											
**Blood NAT**	Pos	Pos		8	6.2		2	Neg							

**IgG/IgM**	Neg/Neg	Neg/Neg						Neg/Neg							
**15**	M-1	D+368	D+3	Ribavirin: 52 d											
**Blood NAT**	Neg	6.63		6.63	2.42		Neg								
**IgG/IgM**	Pos/Q	Nd													
**16**	M-1	D+98	D+5	Ribavirin: 345d											
**Blood NAT**	Nd	7.83		7.83	3.15	2.92	Neg		Neg				Neg		Neg
**IgG/IgM**	Neg/Neg	Neg/Neg		Neg/Neg									Pos/Neg		Neg/Neg
**21**	M-1	D+26	D+1	Ribavirin: 107 d											
**Blood NAT**	Neg	5.43		5.62	3.43	Neg	Neg	Neg	Neg						
**IgG/IgM**	Neg/Neg	Neg/Neg													
**22**		D+120	D+229	Ribavirin: 97 d											
**Blood NAT**	Nd	Pos		Pos		Neg		Neg	Neg						
**IgG/IgM**	Nd														
**24**	D-21	D+52	D+10	Ribavirin: 92 d											
**Blood NAT**	5.97	8.43		8.43					Neg						
**IgG/IgM**	Nd/Nd	Neg/Neg		Neg/Neg					Pos/Pos						
**25**		D+1926	D+23	Ribavirin: 90 d											
**Blood NAT**	Nd	5				Neg									
**IgG/IgM**	Nd	Neg/Neg													

Abbreviations: D: days; NAT: nucleic acid testing; Neg: negative; Nd: not done; Pat: patient; Pos: positive; Q: questionable; Tx: transplantation.

HEV NAT is indicated in log

Supplementary figures legends

SFig1. HEV infection cases diagnosed before alloHSCT

Days are indicated from transplantation

SFig2. HEV infection recurrence after immunosuppressive treatment for graft-versus-host-disease

Days are indicated from transplantation

SFig1. HEV infection cases diagnosed before alloHSCT

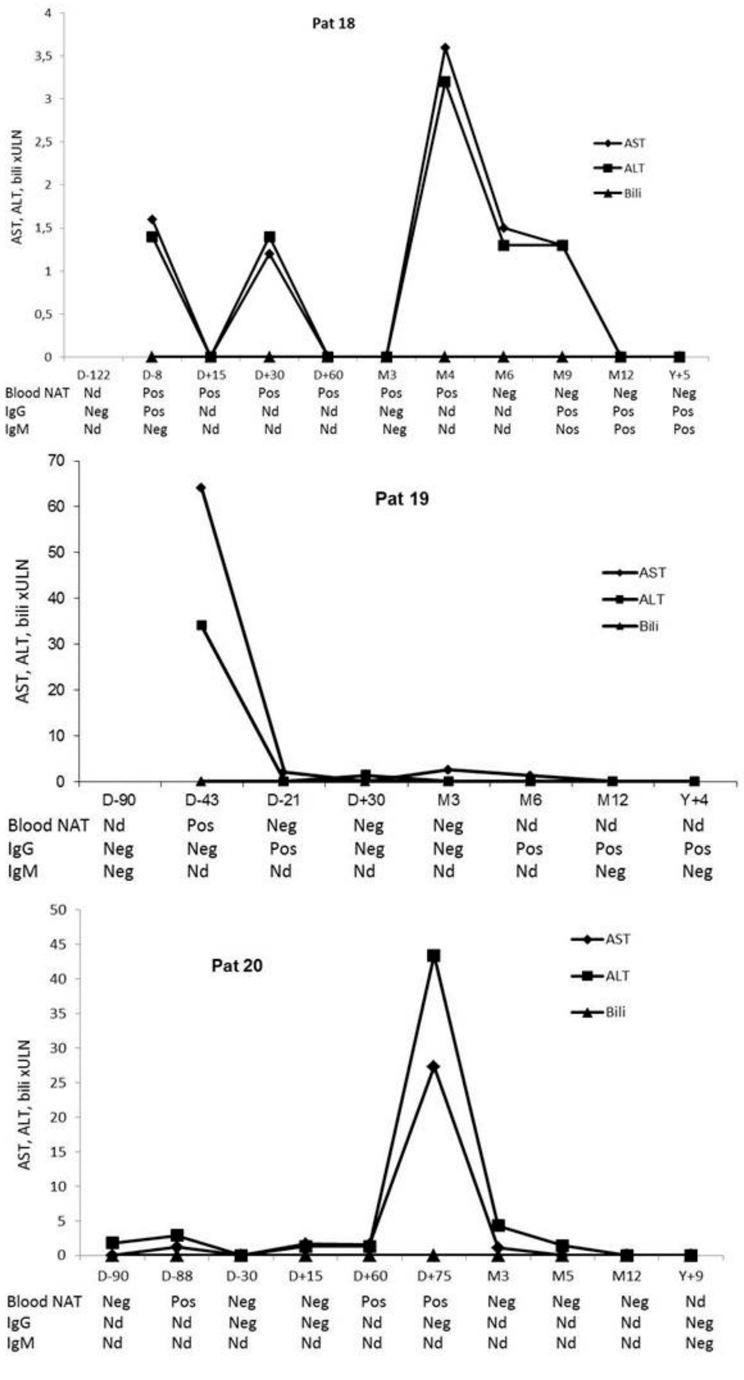

SFig2. HEV infection recurrence after immunosuppressive treatment for graft-versus-host-disease

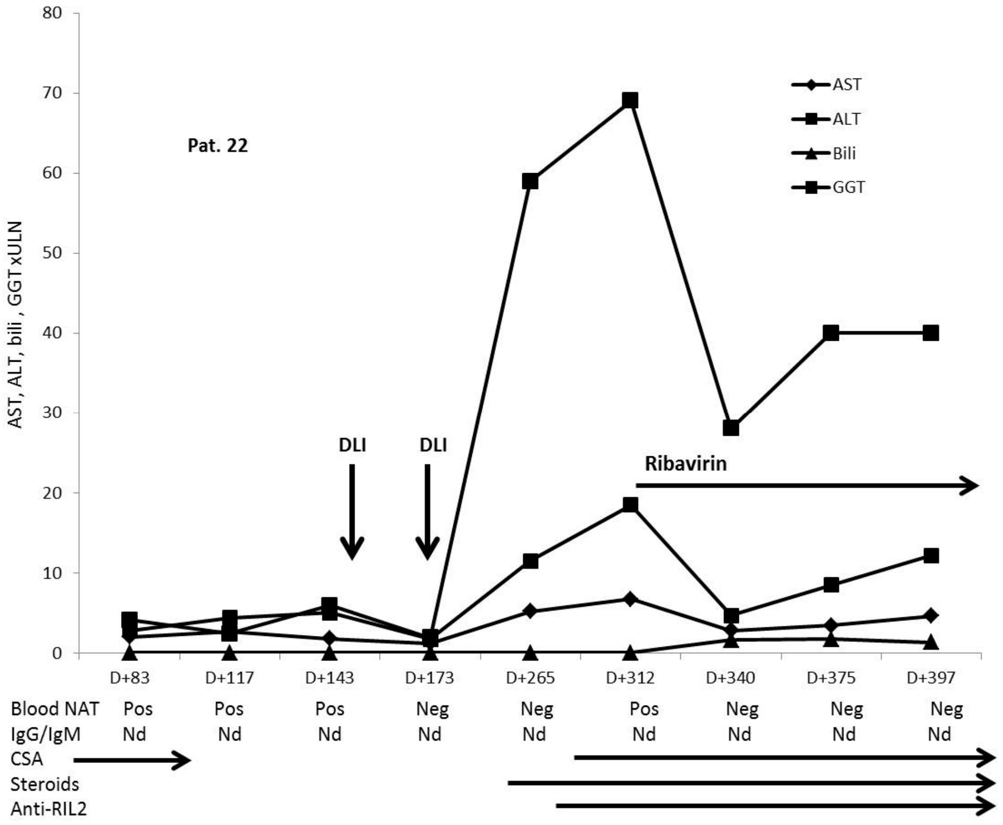

Supplement: Supplementary file 1 [file viruses-11-00622-s001.pdf]
